# BATS: a Bayesian user-friendly software for Analyzing Time Series microarray experiments

**DOI:** 10.1186/1471-2105-9-415

**Published:** 2008-10-06

**Authors:** Claudia Angelini, Luisa Cutillo, Daniela De Canditiis, Margherita Mutarelli, Marianna Pensky

**Affiliations:** 1 Istituto per le Applicazioni del Calcolo, 'Mauro Picone', CNR-Napoli, Italy; 2 Telethon Institute of Genetics and Medicine, Napoli, Italy; 3 Istituto per le Applicazioni del Calcolo, 'Mauro Picone', CNR-Roma, Italy; 4 Dipartimento di Patologia generale, Seconda Università di Napoli, Italy; 5 Department of Mathematics, University of Central Florida, USA

## Abstract

**Background:**

Gene expression levels in a given cell can be influenced by different factors, namely pharmacological or medical treatments. The response to a given stimulus is usually different for different genes and may depend on time. One of the goals of modern molecular biology is the high-throughput identification of genes associated with a particular treatment or a biological process of interest. From methodological and computational point of view, analyzing high-dimensional time course microarray data requires very specific set of tools which are usually not included in standard software packages. Recently, the authors of this paper developed a fully Bayesian approach which allows one to identify differentially expressed genes in a 'one-sample' time-course microarray experiment, to rank them and to estimate their expression profiles. The method is based on explicit expressions for calculations and, hence, very computationally efficient.

**Results:**

The software package BATS (Bayesian Analysis of Time Series) presented here implements the methodology described above. It allows an user to automatically identify and rank differentially expressed genes and to estimate their expression profiles when at least 5–6 time points are available. The package has a user-friendly interface. BATS successfully manages various technical difficulties which arise in time-course microarray experiments, such as a small number of observations, non-uniform sampling intervals and replicated or missing data.

**Conclusion:**

BATS is a free user-friendly software for the analysis of both simulated and real microarray time course experiments. The software, the user manual and a brief illustrative example are freely available online at the BATS website:

## Background

Gene expression levels in biological systems can be influenced by different stimuli, e.g. pharmacological or medical treatments. The response is a dynamic process, usually different for different genes. One of the goals of modern molecular biology is the high-throughput identification of genes associated with a particular treatment or a biological process of interest. The recently developed microarray technology allows one to simultaneously monitor the expression levels of thousands of genes, thus providing a "molecular picture" of a biological system under study and a potential of describing evolution of gene expressions in time. However, this potential has not yet been fully exploited since there is still a shortage of statistical methods which take into account the temporal relationship between the samples in microarray analysis. In fact, most of the existing software packages essentially apply techniques designed for static data to time-course microarray data. For example, the SAM software package (see [1]) was recently adapted to handle time course data by regarding the different time points as different groups. The ANOVA approach by [2] was applied to time course experiments by treating the time variable as a particular experimental factor. Papers by [3,4] and the Limma package by [5] have similar approaches.

All these methods can still be very useful when very short time course experiments have to be analyzed (up to about 4–5 time points), however the shortcoming of these approaches is that they ignore the biological temporal structure of the data producing results that are invariant under permutation of the time points. On the other hand, most classical time series or signal processing algorithms have rigid requirements on the data (high number of time-points, uniform sampling intervals, absence of replicated or missing data) which microarray experiments rarely meet. The past few years saw new developments in the area of analysis of time-course microarray data (see e.g. [6,7], and more comprehensive approaches of [8,9], and [10], implemented respectively in the software EDGE [11] and in the R-packages maSigPro and *timecourse*).

In what follows, we present BATS (Bayesian Analysis of Time Series), a user-friendly software package which implements a novel, truly functional and fully Bayesian approach of [12], specifically designed for the analysis of 'one sample' time series microarray data. Similarly to the other functional approaches to time course data (see, [8,13] and [14]), the proposed method is particularly suitable for time course experiments where at least 5–6 time points are available. Presence of replicated measurements is recommended, but not required.

The software allows an user not only to automatically identify and rank differentially expressed genes, but also to estimate their expression profiles. The latter feature allows an user, for each differentially expressed gene, to visualize its response to the treatment in the course of time as a single smooth curve and, hence, to reveal important biological features that can be hidden in the raw data. The estimates of gene expression profiles are, in fact, more robust than the classical straight-line connecting of the raw data and allow to compare responses of genes to treatment at any arbitrary time point. The truly functional approach of BATS successfully manages various technical difficulties such as non-uniform sampling intervals and replicated or missing data.

### Methodology

The present version of BATS is designed for the analysis of 'one sample' time series microarray data. The name 'one sample' refers to all microarray data where the problem can be formulated in terms of analysis of a single time series. Such kind of data can be obtained, for example, by direct hybridization of the samples corresponding to two biological conditions (e.g., treated and control) and measuring relative expression values on a time grid. Thus, in a 'one sample' problem the data consists of the records, for *N *genes, of the differences in gene expression levels between the sample of interest and a reference (i.e., treated and control) in the course of time. Each record is modeled as a noisy measurement of a function *s*_*i*_(*t*) at a time point *t*^(*j*) ^∈ [0, *T *] which represents the differential gene expression profile:

(1) $$z_{i}^{j,k}=s_{i}(t^{\left(j\right)})+\zeta_{i}^{j,k},i=1,...,N,j=1,...,n,k=1,...,k_{i}^{\left(j\right)}.$$

Here the number *N *of time points is relatively small, with very few replications available at each time point ($k_{i}^{\left(j\right)}$ = 0,...,*K*), while the number *N *of genes is very large, and a total of $M_{i}=\sum_{j=1}^{n}k_{i}^{\left(j\right)}$ observations are available for each gene. The objective is to identify the genes showing different functional expression between treated and control (i.e. *s*_*i*_(*t*) ≠ 0), and then to evaluate the effect of the treatment (i.e., estimate the curves *s*_*i*_(*t*)).

For each gene *i*, we expand its expression profile *s*_*i*_(*t*) into series over some standard orthonormal basis on [0, *T*] with coefficients $c_{i}^{\left(l\right)}$, *l *= 0,...,*L*_*i*_:

(2) $$s_{i}(t)=\sum_{l=0}^{L_{i}}c_{i}^{\left(l\right)}\phi_{l}(t).$$

Legendre polynomials and Fourier basis suitably rescaled and normalized in [0, *T*] are supported in the current version of BATS.

Following [12], genes are treated as conditionally independent and their expressions are modeled as **z**_*i *_= **D**_*i*_**c**_*i *_+ ***ζ***_*i*_. Here, **D**_*i *_is the block design matrix, the *j*-row of which is the block vector $\left[\phi_{0}(t^{\left(j\right)})\phi_{1}(t^{\left(j\right)})\ldots \phi_{L_{i}}(t^{\left(j\right)})\right]$ replicated $k_{i}^{\left(j\right)}$ times; $z_{i}={\left(z_{i}^{1,1}\ldots z_{i}^{1,k_{i}^{\left(1\right)}},\cdots ,z_{i}^{n,1},\ldots z_{i}^{n,k_{i}^{\left(n\right)}}\right)}^{T}$, $c_{i}={\left(c_{i}^{\left(0\right)},\ldots ,c_{i}^{\left(L_{i}\right)}\right)}^{T}$ and $\zeta_{i}=\zeta_{i}^{1,1},\ldots ,\zeta_{i}^{1,k_{i}^{\left(1\right)}},\cdots ,\zeta_{i}^{n,1},\ldots ,\zeta_{i}^{n,k_{i}^{\left(n\right)}}{)}^{T}$ are, respectively, the column vectors of all measurements for gene *i*, the coefficients of *s*_*i*_(*t*) in the chosen basis and random errors. The following hierarchical model is imposed on the data:

$$\begin{array}{cc} \begin{array}{r} z_{i}|L_{i},c_{i},\sigma^{2}\\ L_{i}\\ c_{i}|L_{i},\sigma^{2} \end{array} & \begin{array}{ll} \sim & N(D_{i}c_{i},\sigma^{2}I_{M_{i}})\\ \sim & \text{Truncated Poisson }(\lambda ,L_{\max})\\ \sim & \pi_{0}\delta (0,...,0)+(1-\pi_{0})N(0,\sigma^{2}\tau_{i}^{2}Q_{i}^{-1}) \end{array} \end{array}$$

All parameters in the model are treated either as random variables or as nuisance parameters, recovered from the data. Noise variance *σ*^2^is assumed to be random, *σ*^2 ^~ *ρ*(*σ*^2^) in order to account for possibly non-Gaussian errors, quite common in microarray experiments. Currently, BATS supports three types of priors:

**Model 1: ***ρ*(*σ*^2^) = *δ*(*σ*^2 ^- $\sigma_{0}^{2}$), the point mass at $\sigma_{0}^{2}$. The marginal distribution of the error is normal.

**Model 2: ***ρ*(*σ*^2^) = *IG*(*γ*, *b*), the Inverse Gamma distribution. The marginal distribution of the error is Student *T*.

**Model 3: ***ρ*(*σ*^2^) = $c_{\mu }\sigma^{M_{i}-1}e^{-\sigma^{2}\mu /2}$. The marginal distribution of the error is double exponential.

An automatic detection of differentially expressed genes is carried out on the basis of Bayes Factors (*BF*), which are used for taking into account multiplicity of errors. This technique is based on the novel methodology of [15] which is similar in spirit to the procedure of [16] for controlling the False Discovery Rate (FDR).

Once the differentially expressed genes have been detected, the coefficients $c_{i}^{\left(l\right)}$ and, subsequently, the curves *s*_*i*_(*t*) are estimated by the posterior means.

The algorithm is self-contained. The hyperparameters *π*_0 _and $\sigma_{0}^{2}$, (*γ*, *b *or *μ *for Model 2 or Model 3, respectively) are estimated from the data (several procedures are available), or they can be entered as known by an user. Gene specific parameters $\tau_{i}^{2}$ are estimated by maximizing the marginal likelihoods, while *L*_*i *_are estimated by the posterior mean or mode. Explicit formulae and other details can be found in [12].

A great advantage of the Bayesian model described above is that all evaluations are carried out in analytic form (see [12] for details), with very efficient computations.

**Remark 1 ***BATS implements a truly functional Bayesian approach. Hence, by construction, it is designed for those time-course experiments where at least 5–6 time points are available, although in order to fully exploit the advantage of the functional approach a somewhat larger number of time points and of arrays is recommended. A sharp limit is hard to elicit, since the decision depends also on whether the replicates are available, on the type of the grid design and on the biological assumptions on the process under investigation. In principle, BATS can also be used with fewer than 5 time points, but in that case no particular gain is guaranteed with respect to a classical regression based approach. However, we point out that similar requirements are typical to the other functional data approaches*.

### Algorithm

The algorithm is performed by carrying out the following steps:

1. Choose the prior parameters *λ*, *L*_max _and *ν*, fix the type of the orthonormal basis that will be used in the analysis.

2. Estimate global parameters: *σ*^2 ^and *π*_0_, and additional case-specific hyper-parameters $\sigma_{0}^{2}$ (for MODEL 1), *γ *and *b *(for MODEL 2) or *μ *(for MODEL 3). Several options are provided to an user, including the possibility of custom definition of parameters.

3. For each gene *i*, estimate the gene specific parameter $\tau_{i}^{2}$ by maximizing the marginal pdf of the data.

4. For each gene *i*, estimate the degree of polynomial *L*_*i *_by the posterior mean or the posterior mode.

5. For each gene *i*, conditionally on ${\hat{L}}_{i}$, compute Bayes Factor *BF*_*i*_.

6. Perform the Bayesian multiple testing procedure of [15] to rank the genes according to the ordered Bayes Factors. The user can choose to automatically determine a cut-off of significance according to different priors or to simply order the genes.

7. Estimate the gene expression profiles by ${\hat{s}}_{i}(t)$ substituting the posterior mean estimator of **c**_*i *_in (2).

### Remark 2

*Since all evaluations in BATS are carried out in analytic form, the computational cost remains very moderate. The N gene-per-gene one-dimensional maximization in Step 3 represents the most computationally demanding part of the algorithm. The results of the analysis up to 50.000 probes and 25 arrays is usually returned in 20 minutes using the compiled Windows version on a Pentium IV PC computer with 3.00 GHz and 2 GB of RAM, the exact time depending on the total number of probes and the distributions of missing data*.

*In principle all the probes available on the arrays can be analyzed. However, from a practical point of view, probes containing too many missing values should be removed from the analysis since they may not carry reliable information. Similarly, control probes or probes which are not expressed can be removed if information which they carry is not considered significant or of biological interest*.

## Implementation

BATS is a graphical user-friendly software written in MATLAB. Executable program for Windows, Linux and Mac Osx, the source code and the user manual can be freely downloaded from .

Permission to use, copy, modify, and distribute BATS for any purpose without fee is granted by the BATS permissive license (derived from the MIT license). The compiled software needs to run the MATLAB component Runtime (MCR), also available on the website for the sole purpose of running BATS.

**Figure 1:**
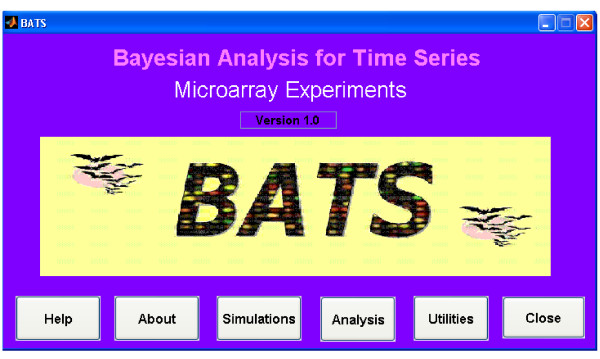
The Main Menu of BATS.

Current implementation of BATS is designed for a single processor, and it is fast enough for any practical purpose. Version 1.0 of BATS is composed of two main applications: ANALYSIS and SIMULATIONS; it is equipped with a third option, UTILITY, which provides additional functions. Each application can be activated from the main window (see Figure 1).

A context-specific HELP button is present in all windows, providing all necessary information as well as a short description of all the parameters required by a procedure. The ABOUT button reports the Terms of the License. A more detailed description can be found in the USER REFERENCE MANUAL. The guided TUTORIAL available on the website can be used for a fast introduction to the software. In what follows, we briefly describe each application.

### Analysis

**Figure 2:**
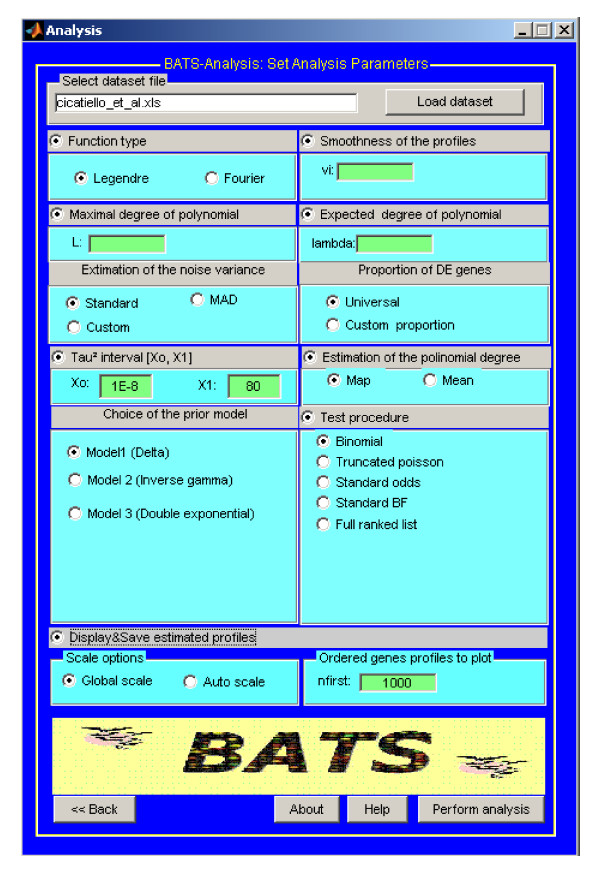
**The Analysis window of BATS**.

The ANALYSIS application allows to apply the methodology developed in [12] to either synthetic or real data-sets. The menu of ANALYSIS application is divided into sub-windows (see Figure 2) which allow an user to define the parameters of the analysis. Obviously, ANALYSIS constitutes the most important part of BATS from biologists' point of view.

Data can be loaded into the system and analyzed on the basis of any of the three error models described in Section "Methodology" and denoted in the software as MODEL 1, MODEL 2 and MODEL 3, respectively. The input data should be in the EXCEL spreadsheet or a tab-delimited text file format prepared as follows. The first row should contain a text string (i.e., GENE NAME) in the first column, and, in the remaining columns, the numerical values of the time measurements *t*^(*j*) ^in ascending order and represented in the same time units (seconds, hours, days, etc.). From the second row on, the first column should contain the gene identifier, a unique string of letters or a combination of letters and numbers (numbers only are not allowed). The remaining columns should contain data, $z_{i}=(z_{i}^{1,1}\ldots z_{i}^{1,k^{\left(1\right)}},\cdots ,z_{i}^{n,1},\ldots z_{i}^{n,k^{\left(n\right)}})$), in the form of log_2_-signal-to-reference ratios. Missing values can be entered as either empty cells or NaN. Before analyzing microarray data with BATS, the data should be pre-processed to remove systematic sources of variation. For a detailed discussion of the normalization procedures for microarray data we refer the reader to e.g., [17-19] or [20]. We recall (see also Remark 1) that BATS is particularly suitable for those experiments where at least 5–6 different time points are available. Moreover, although BATS automatically accounts for missing data, for a reliable analysis we suggest that the proportion of missing data should remain relatively small (for each gene at least 50–60% of the observations should be available). Note, if the data set to be analyzed does not meet these general requirements, a warning message will be displayed. From the ANALYSIS window, an expert user can choose prior parameters (see Step 1 of the Algorithm). We briefly discuss these choices below. A detailed description can be found in the user-manual.

The type of basis functions can be either Legendre or Fourier, with default choice Legendre. The global regularity *ν *of the gene expression profiles is a real number in [0, 1], (default value 0). The maximum degree *L*_max _allowed in the expansion is an integer value, default value [*n/*2] as a compromise between the goodness of fit and variance of the estimate. The parameter *λ *of the Poisson distribution truncated at *L*_max _has to be chosen in order to match the prior expected degree of the polynomial.

Choosing appropriate parameters for the analysis of a particular data-set with BATS usually requires some preliminary knowledge of statistics and some level of expertise. However, a user who is not an expert in statistics should not be discouraged, since for all parameters BATS provides default values that can be used in most cases, and the parameters' sub-windows are hidden by default. If necessary, hidden windows can be opened in order to change the default values.

After that, the user can either select a specific method for estimating global parameters *π*_0 _and *σ*_0_, or enter their values manually by choosing the CUSTOM option (see Step 2 of the Algorithm). In the current version of BATS, estimation of the global parameters is based only on the *N*_*c *_genes for which the complete set of *M *observations is available. If the default option STANDARD remains selected, for each array of observations at a time point *t*^(*j*)^, *σ*^(*j*) ^is estimated by the sample standard deviation ${\hat{\sigma }}^{\left(j\right)}$. On the other hand, if normal distribution of the data can be justified, by selecting the corresponding option MAD, the sample variance can be replaced by a more robust estimator like the Median Absolute Deviation, which is usually proposed when the majority of array components are zeros [21]. In both cases, the estimator ${\hat{\sigma }}^{2}$ is obtained by averaging of (${\hat{\sigma }}^{\left(j\right)}$)^2^, *j *= 1,..,*M*.

Given $\hat{\sigma }$, with the option UNIVERSAL, following [21], the global parameter *π*_0 _is estimated by averaging over the arrays the proportion of data points which fall below the universal threshold $\hat{\sigma }\sqrt{2\log N_{c}}$. Note that this method tends to overestimate *π*_0 _when the error is normally distributed, but not when the error distribution has heavier tails, which is very common in microarray data.

Once one of the three error models has been selected in the box CHOICE OF THE PRIOR MODEL, the model-dependent parameters are estimated automatically for MODELS 1 or 3. If MODEL 2 is selected, the user can further choose the way for estimating the hyperparameters *b *and *γ*. Specifically, with CHOICE 2, *γ *and *b *are estimated by using the Maximum Likelihood Estimator (MLE) on the set of values ${\hat{\sigma }}^{\left(j\right)}$, *j *= 1,...,*M*, which are treated as a sample from the distribution of *σ *(note that if (${\hat{\sigma }}^{\left(j\right)}$)^2 ^~ *IG*(*γ*, *b*), then (${\hat{\sigma }}^{\left(j\right)}$)^-2 ^~ Gamma(*γ*, *b*)). If the user selects alternative option CHOICE 1, he/she has to fix *γ *and then parameter *b *will be automatically evaluated by matching the mean of *IG*(*γ*, *b*) with ${\hat{\sigma }}^{2}$. We observe that with selection of CHOICE 2 an user does not have to specify any parameters. With CHOICE 1, an user have to specify the positive parameter *γ *(default value 15). The two options produce slightly different lists of genes and allow to check the robustness of the selections.

An user can also choose whether to estimate the degree of the polynomial *L*_*i *_by the posterior mean (option MEAN) or the posterior mode (option MAP) (Step 5 of the Algorithm) from the box ESTIMATION OF THE POLYNOMIAL DEGREE, and what procedure to use for testing which of the genes are differentially expressed (Step 6 of the algorithm) from the box TEST PROCEDURE. In the latter, the default option BINOMIAL refers to the Binomial prior elicited on the number of alternative hypotheses, option TRUNCATED POISSON (with further choices which of the stepwise approaches to use in order to decide which hypothesis to accept and which to reject, see [15] for details) is based on the truncated Poisson prior. Options STANDARD ODDS, STANDARD BF do not implement any multiplicity control and option FULL RANK only ranks the genes without providing any automatic cut off.

An user has an option to print out the estimated profiles (superimposed to the raw data) for the top 'nfirst' genes according to ranking, either in 'Global scale' (all gene profiles are shown on the same scale to make the figures comparable) or in 'Auto scale' (each gene profile is shown using the most appropriate scale in order to improve visualization). We note that visual inspection of the profiles can be very useful for a quick assessment of the fit.

Alternatively, expression profiles of individual genes can be generated later using the Utility – PLOT PROFILES.

Once the necessary parameters have been defined, an user has to choose a Project name and launch the analysis. By default, for each run of the analysis, three files are generated in the folder Projects: a summary of the analysis _SR.txt (reporting all the parameters used), the ordered list of differentially expressed genes _GL.xls for Windows systems or _GL.txt for Linux or Mac Osx, and the estimated gene profiles _SH.xls for windows systems or _SH.txt for Linux or Mac Osx. The dialog window shows intermediate results and stages of the algorithm during the execution of the analysis.

### Simulations

The SIMULATIONS application enables an expert user to generate, analyze and save synthetic data. This feature can be useful for planning experimental design (e.g., for finding an acceptable balance between the cost and the benefits of increasing the number of arrays, for deciding whether to employ new arrays as further replicates at existing time points or at additional time points), for preliminary verification whether BATS is a suitable tool for a given type of experiments, or for generating synthetic data which can be used for comparison of other statistical tools. This application can also be used to enhance understanding of some features of the proposed software. Simulations are indeed a typical tool for validation and comparisons of statistical procedures. They are also widely used in microarray analysis, see, for example, [9,10] and [13]. Running an appropriate simulation study requires some basic knowledge of statistics and some experience in computing.

**Figure 3:**
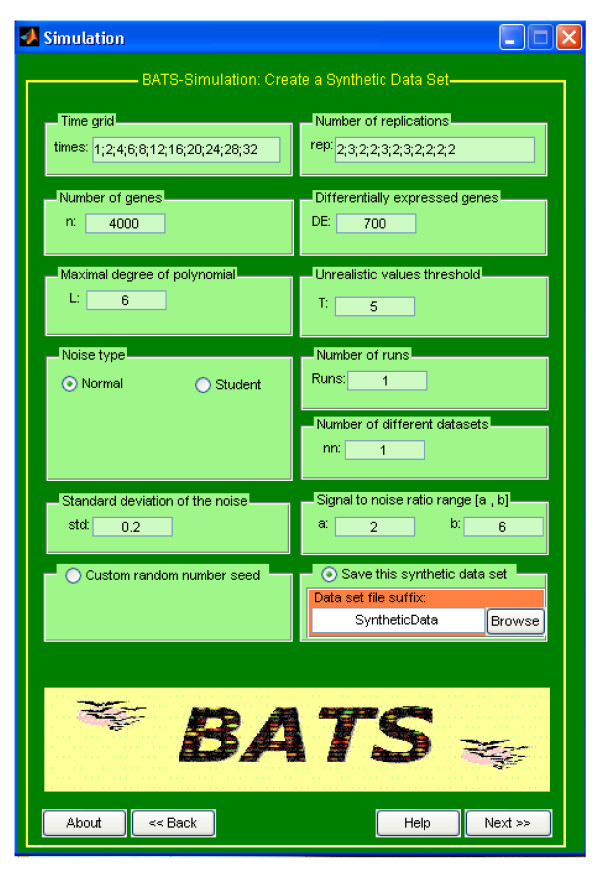
The first Simulation window of BATS.

The SIMULATIONS application consists of two windows. In the first window (see Figure 3) an user provides parameters required to generate synthetic data. In the second window the user can choose how to analyze the generated data set (the second window is similar to the ANALYSIS window).

Synthetic data-sets can be generated and saved for later use in the original form, or after removing some data. For example, an user may decide to generate data using a very fine time grid and after that to analyze them using only a sub-set of the synthetic arrays or by randomly replacing some synthetic values with missing numbers. The simulated data are recorded in the BATS-input format with an extra sheet or an additional file (sheet2 in the .xls format or an additional file in the .txt format) containing the flags which are set to one for the genes which are differentially expressed and to zero for those which are not. Synthetic data-sets can also be used to compare performance of BATS with other available methods as it is done in [12].

In the process of generating data-sets, an user has to choose the following parameters: the total number of genes *N*, the number of differentially expressed genes *DE*, the time grid *t*^(*j*)^, *j *= 1,...,*n*, and the maximum number of replications *k*^(*j*) ^at each time point *t*^(*j*) ^(in principle, such information should be provided by a biologist). For each significant curve, the algorithm first samples the degree of the polynomial $L_{i}^{true}$ from a discrete uniform distribution in [1, *L*_max_]. Polynomials of degree zero are avoided since a nonzero constant signal is questionable from a biological point of view. After that, for each gene *i*, a vector of coefficients **c**_*i *_is randomly sampled from the multivariate normal distribution $N(0,\sigma^{2}\tau_{i}^{2}Q_{i}^{-1})$ where the experimental variance *σ*^2 ^is chosen by the user (on the basis of user's experience and other available information). Matrix **Q**_*i *_is set to be **Q**_*i *_= diag $\left(1^{2\nu_{i}},2^{2\nu_{i}},...,L_{i}^{2\nu_{i}}\right)$ where *ν*_*i *_~ *U*([0, 1]). An user can also choose the range from where the gene specific variance $\tau_{i}^{2}$ is randomly sampled. For this purpose, from the box SIGNAL TO NOISE RATIO RANGE the user can choose parameters *a *and *b *such that $\tau_{i}^{2}$ is sampled uniformly in order to produce the signal-to-noise ratio (SNR) in [*a*, *b*].

Synthetic data-sets are generated according to the model (1) by adding i.i.d noise to the simulated profiles. Two types of noise distributions are supported in the current version of BATS: normal *N*(0, *σ*^2^) and Student *T *with at least 3 degrees of freedom. In order to make results of several simulation comparable, Student noise is scaled to have the same variance *σ*^2 ^as in the normal case. In addition, setting a threshold T in the box THRESHOLD FOR UNREALISTIC VALUES forces simulated values larger than T to be filtered out and replaced with "missing values", mimicking pre-processing of real data where unrealistic values are eliminated.

**Figure 4:**
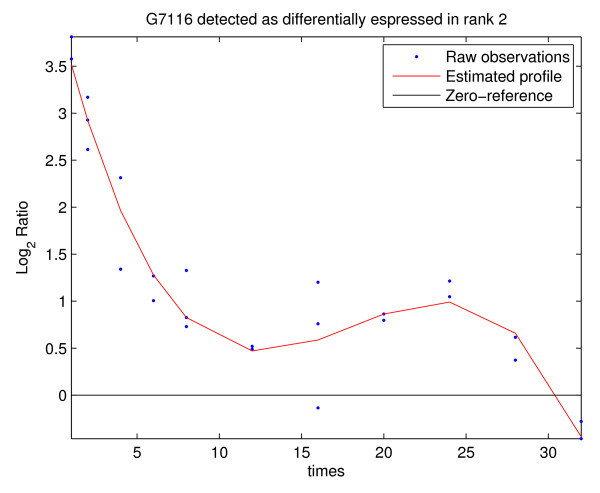
**Example of simulated gene expression profile**. The profile is a significant synthetic profile generated by choosing the time observations on the grid 1, 2, 4, 6, 8, 12, 16, 20, 24, 28 and 32 hours; using two replicates for each time point and three replicates at *t *= 2, 8, 16), the values of the other parameters were *N *= 8000, *D *= 600, *L*_max _= 6, *λ *= 9, *ν *= 0, σ= 0.3, *SNR *= [2, 6], the noise affecting the data was *T *(5).

The simulation scheme is similar to the one proposed in [13]. If the parameters of the simulated data are chosen correctly, the synthetic profiles should resemble the true raw data. Synthetic profiles can be displayed using the utility PLOT PROFILES and visually inspected in order to assess their biological resemblance. In Figure 4 a synthetic profile is shown. The profile was generated choosing the time observations on the grid 1, 2, 4, 6, 8, 12, 16, 20, 24, 28 and 32 hours with two replicates for each time point and three replicates at *t *= 2, 8, 16); the values of the other parameters were *N *= 8000, *D *= 600, *L*_max _= 6, *λ *= 9, *ν *= 0, *σ *= 0.3, *SNR *= [2,6], the noise affecting the data was *T*(5). It should be noticed that synthetic data can only provide basic suggestions about the performance of BATS since real data often has complex structure which is very hard to model precisely in mathematical terms.

Using the same simulation set-up, several data-sets can be created with several randomly generated sets of profiles *s*_*i *_and several different noise realizations. Each synthetic data-set can be analyzed assuming any of MODEL 1, 2 or 3.

Performance of the technique is automatically evaluated using the False discovery rate (FDR), False negative rate (FNR), the numbers of correctly detected, not detected or misclassified genes and some other standard measures (e.g., functional estimation errors). The results are automatically averaged in order to provide statistically relevant information which is not dependent on a particular random realization. An output .txt file contains the results of the analysis, while the dialog window shows intermediate messages during computations.

### Utilities

**Figure 5:**
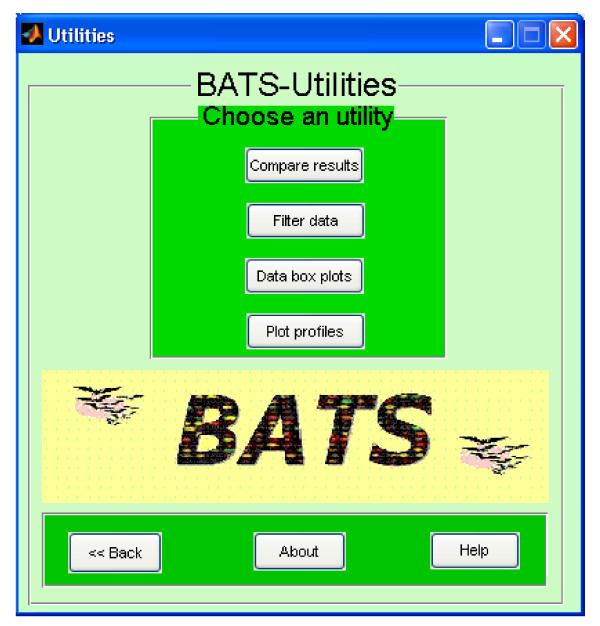
The Utilities menu of BATS.

The UTILITIES menu (see Figure 5) provides a set of procedures FILTER DATA, DATA BOX PLOTS, COMPARE RESULTS and PLOT PROFILES that help an user to process and visualize input or output files. Other utility functions will be added to future versions of BATS.

The procedure FILTER DATA can be used to remove genes with a number of missing measurements larger than a desired threshold before starting the analysis (see Remark 2). A new BATS input data file will be created containing the filtered data.

**Figure 6:**
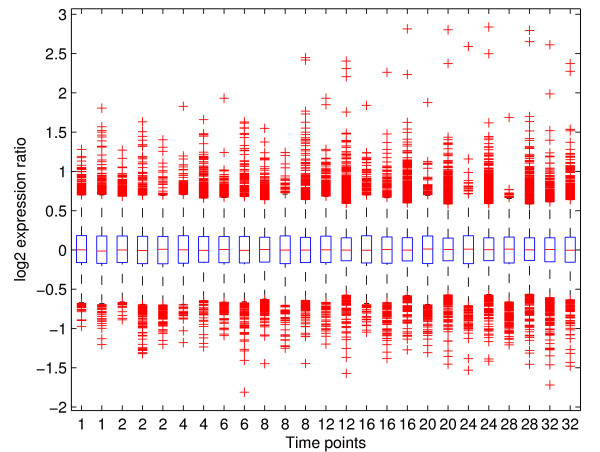
**Boxplots of the *log*_2 _expression ratios in the experiment described in**[23]. The Data-set is included as an example in BATS.

Similarly, the utility DATA BOX PLOTS can be used to compactly represent data for inspection before starting the analysis (Figure 6). For each array the boxplot shows the median of all values (central red lines), the range which covers 50% of values (blue boxes), the range which covers 75% of values (dashed black lines) and all the remaining individual values (red crosses). Normalized array values should all have the same median and also approximately the same range of values. Additional information about the experiment such as the total number of missing values, the number of missing values per gene and per array are displayed in the dialog box.

After a series of analyses have been performed on the same data-set using different parameters or models, the utility COMPARE RESULTS allows an user to easily compare the results stored in _GL.xls (or _GL.txt) files and, hence, investigate the robustness of the lists of the differentially expressed genes. Two files are created by this option: a _common.xls (or _common.txt) file containing the intersection of all the selected lists and reporting for each gene the rank obtained in each analysis, and a _union.xls (or _union.txt) file containing all the genes present in at least one of the lists.

Finally, the function PLOT PROFILES provides an alternative way to visualize the data and the selected gene expression profiles. For this purpose, an user can choose whether to plot the raw data or the expression profiles for differentially expressed genes, or both. The input data-set needs to be loaded from the sub-window Select a raw data file name together with the name of the file (i.e., the _SH.xls or _SH.txt file) which contains estimated expression profiles resulted from the previous analysis, if the profiles of the differentially expressed genes need to be plotted. Then, the list of all genes in the files is shown, and the user can select the genes of interest. Additionally, the user can choose some plotting options such as the color of the line or the type of the marker. The corresponding individual profiles are displayed sequentially, and the plots can be saved as image files.

## Results

The statistical method implemented in BATS has been validated using both real and simulated data in [12] and [22]. The performance of BATS has also been compared with two recent competitive methods: [8] and [10]. The first method is implemented by the EDGE software [11] while the second by the R-package *timecourse *(see [12] and [22] for a detailed discussion).

In the following, in order to illustrate the benefits of using BATS, first, we summarize the results of its application to the real data set contained in the Examples folder in BATS and used in the tutorial for a guided analysis, then we compare the findings with the ones obtained using EDGE and *timecourse *on the same data-set.

We note that since all three methods apply to different experimental designs, account for different biological information and are valid under different assumptions, we felt that it would be more fair to compare our method with the others using a real data set that does not conform to the assumptions in the present paper.

The data-set refers to the experiment described in [23]. In the experiment, human breast cancer cell line ZR-75.1 cultures were stimulated with 5·10^-8^*M *17*β*-estradiol (E2) after being maintained for 4 days in steroid-free medium. RNA samples were extracted before the stimulation and after 1, 2, 4, 6, 8, 12, 16, 20, 24, 28 and 32 hours of stimulation. The cDNA microarray analysis was carried out with Human UniGEM V 2.0 glass arrays (Incyte Genomics, St Louis, MO, USA). For each time point at least two replicates were available (three replicates at *t *= 2, 8, 16).

Complete data can be downloaded from the NCBI public gene expression data repository Gene Expression Omnibus (GEO Acc: GSE186). In this context the results of [23] provides a "biology-guided" selection of significant genes that can be used as a "benchmark" in our comparisons. For a more detailed comparisons including simulated data the reader is referred to [12] and [22].

The data file 'Cicatiello_et_at.xls' contains the relative expression values $z_{i}^{j,k}$ measured as the log_2 _treated to control fluorescence intensity ratio. Data contained in the provided file have been already pre-processed, normalized and presented in the BATS input format.

**Figure 7:**
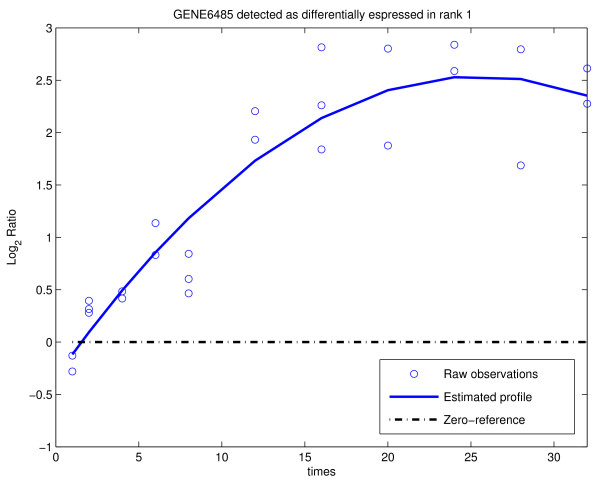
**Gene6485 (TFF1, a well-known target of the estrogen receptor) has been selected with rank 1 by BATS and included in the list of 574 genes selected by all the 28 combinations.** This gene was detected in [23] too.

**Figure 8:**
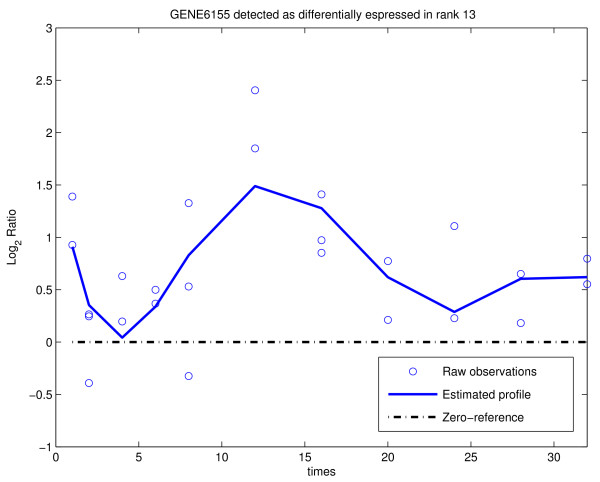
**Gene6155 (MKI67, a gene involved in cell-cycle control but with a less clear association with estrogen action in literature) has been selected with rank 13 by BATS and included in the list of 574 genes selected by all the 28 combinations.** This gene was not detected in [23].

The data set has been analyzed using MODELS 1, 2 and 3 and various combinations of parameters. Different outputs were then compared in order to seek for genes common to all options of the analysis and for those which are selected only under a particular combination of parameters. After each analysis, the list of genes detected as differentially expressed was saved in a project_name_GL.xls file. After several runs of the analysis, the _GL.xls files were compared using the function COMPARE RESULTS in the UTILITY menu. In what follows, we report the results of the analysis with MODELS 1, 2 and 3 and various choices of *λ*. Table 1 displays the number of genes declared affected by the treatment for *L*_max _= 6, *ν *= 0 and *λ *ranging between 6 and 12 (which corresponds to expected prior degree of polynomials from 2.5 to 3.5). It is easy to see that the results are quite robust with respect to the number of detected genes, with smaller *λ *providing larger lists. Using the function COMPARE RESULTS we discovered that the technique is also robust with respect to the list of genes declared differentially expressed: 574 genes were common to all 28 lists (combination of different methods and different parameter values) while 958 genes have been selected in at least one of the 28 lists. A more detailed discussion of the results of the analysis is provided in [12]. The PLOT PROFILE function allows an user to visualize both raw data and estimated profiles. Figures 7 and 8 show an example of a gene expression profile selected as differentially expressed by both BATS and [23] and an example of a gene selected by BATS but not in [23], respectively.

**Table 1:** Total number of genes in the dataset [23] detected as significant by BATS (with *ν *= and Ł_max _= 6)

	*λ *= 6	*λ *= 7	*λ *= 8	*λ *= 9	*λ *= 10	*λ *= 11	*λ *= 12
case-1	867	808	753	712	692	688	691
case-2-I	893	823	765	711	679	657	650
case-2-II	869	810	755	714	694	690	693
case-3	855	786	726	676	640	617	609

Next, for comparative purposes, we applied EDGE and *timecourse *to the same data-set. To be fair, we should mention that functional statistical approach implemented in EDGE was originally designed for the "two-sample" problem following the paper of [8] and afterwards equipped with a special tool to handle the "one-sample" problem. The approach of [10] applies both to the "one-sample" and the "two-sample" problems for classical longitudinal data where replicates are biologically meaningful, however, it is not a functional data approach. In [14] the authors proposed a new functional data approach, but their software is not yet available to the community.

Since the EDGE software does not automatically account for missing values but only suggests a preliminary procedure (K-nearest-neighbors) for filling them in, we repeated the analysis both using this procedure and filtering out genes with missing values. Additionally, EDGE allows an user to choose the degree of the splines or the polynomials common to all genes. We carried out the analysis with different choices for the maximal degree of the polynomials and found out that the results were robust with respect to those choices (we do not report these results here). To estimate the distribution of the statistics under the null hypothesis, EDGE uses a bootstrap approach, thus requiring a high computational effort and appropriate memory resources. We used 1000 permutations in our comparisons and we discovered that the gene selections were robust with different random seeds (only a few different genes). In order to control the multiplicity error, EDGE uses the *q*-values, which we chose to be *q *= 0.05 and *q *= 0.1 in our analysis. *Timecourse *neither allows missing values nor suggests a specific procedure for treating them; moreover, it requires that each time point has the same number of replicates. Thus, in order to apply the method, we filtered out all the genes with missing observations and discarded the third observations which was available at time points t = 2, 8, 16. To be fair, we should mention that since *timecourse *is designed for data where replicates are biologically meaningful. Since dataset [23] contains only technical (indistinguishable) replicates, in our study *timecourse *package could not take advantage of the replicate identification. On the other hand, the information about the time measurements is not used by *timecourse *method. Since the method only provides rank-ordered list of genes (without any automatic cut off point), we performed the comparisons taking the top 500 and 1000 genes in the resulting list.

Table 2 shows the number of genes detected by different procedures and the overlap with the genes detected as significant in the original paper [23]. BATS has a noticeably wider overlap with the "biology guided" selection of significant genes of [23] and most of the genes selected by EDGE, *timecourse *and [23] were also selected by BATS. In fact, 165 out of the 186 genes selected by EDGE and declared significant in [23] and 166 out of the 174 genes common to the 500 top-ranked genes by *timecourse *and [23] were also contained in the list of 574 genes selected with all the combinations of parameters used in BATS. Finally, 138 genes were common to all selections ([23], all versions of BATS, EDGE and *timecourse*).

**Table 2:** Total number of genes declared affected by the treatment and overlap with the biological selection done in [23]

Methods	Selected genes	Overlap
All of the 28 methods in Table 1	574	270
At least one of the 28 methods in Table 1	958	309
Case 1, *λ *= 9 in Table 1 (default choice)	712	295
EDGE with default choices and q = 0.05	767	186
EDGE with default choices and q = 0.1	1178	219
*Timecourse*	500	174
*Timecourse*	1000	215

## Conclusion

This paper describes BATS, a novel statistical user-friendly software specifically designed for time course microarray data. In particular, BATS allows an user to analyze time series microarray experiments having possibly non-Gaussian errors and as few as 5–6 time points per gene, although a modest increase in the number of available time points will produce a significant improvement of the findings. Presence of replicated measurements is recommended, but not required. It is highly computationally efficient, since all calculations are based on analytic expressions. BATS automatically manages irregular experimental design issues, such as non-uniform sampling intervals and missing or replicated data. The method accounts for multiplicity of errors, selects and ranks differentially expressed genes.

Analysis of the human breast cancer data-set from [23] is provided as a guided example and also for comparison of the results with other possible approaches. Although originally designed for handling cDNA microarray experiments, BATS can be used to analyze data produced by using any microarray platform as showed in [22] where the software is applied on a data-set generated with Illumina BeadChips.

Version 1.0 of BATS is designed for the 'one sample' problem. The extension of the statistical model to the 'two sample' case is currently under development, its implementation will be added in future releases.

## Availability and requirements

The BATS software, user manual and illustrated examples can be downloaded from the BATS website.

1. Project Name: BATS (version 1:0)

2. Project home page: 

3. Operating system(s): Windows, Linux, Mac Osx

4. Programming language: MATLAB

5. Other requirements: 512 MB RAM, 2.0 GHz Pentium 4 CPU, 300 MB free disk space on hard drive, MATLAB Component Runtime (available from the software web site).

6. License: BATS permissive license (derived from the MIT license)

## Authors' contributions

All authors participated in writing the code for the software package, developing the project website, the documentation, and writing the manuscript. All authors also read and approved the submitted manuscript.
